# Effect of Nano-SiO_2_ on Different Stages of UHMWPE/HDPE Fiber Preparation via Melt Spinning

**DOI:** 10.3390/polym15010186

**Published:** 2022-12-30

**Authors:** Qun Yang, Run Zhang, Mingfei Liu, Ping Xue, Lichao Liu

**Affiliations:** College of Mechanical and Electrical Engineering, Beijing University of Chemical Technology, Beijing 100029, China

**Keywords:** UHMWPE, fiber, melt spinning, nano-SiO_2_, crystallization

## Abstract

Ultra-high molecular weight polyethylene (UHMWPE)/high-density polyethylene (HDPE) blend with lower viscosity is more suitable for melt spinning compared to pure UHMWPE; however, the mechanical property of the blend fiber is hard to dramatically improve (the maximum tensile strength of 998.27 MPa). Herein, different content modified-nano-SiO_2_ is incorporated to UHMWPE/HDPE blend fiber. After adding 0.5 wt% nano-SiO_2_, the tensile strength and initial modulus of UHMWPE/HDPE/nano-SiO_2_ fiber are increased to 1211 MPa and 12.81 GPa, respectively, 21.57% and 43.32% higher than that of UHMWPE/HDPE fiber. Meanwhile, the influence of the nano-SiO_2_ content on the performance for as-spun filament and fiber are emphatically analyzed. The crystallinity and molecular chain orientation of as-spun filament reduces with the addition of nano-SiO_2_. On the contrary, for fiber, the addition of nano-SiO_2_ promoted the crystallinity, molecular chain orientation and grain refinement more obvious at a lower content. Furthermore, the possible action mechanism of nano-SiO_2_ in the as-spun filament extrusion and fiber hot drawing stage is explained.

## 1. Introduction

Due to its startling mechanical properties, high wear resistance, excellent corrosion resistance and self-lubricating properties [1,2,3], ultra-high molecular weight polyethylene (UHMWPE), one of the main artificial high-performance fibers, has been used in aerospace [4], military [5], medical [6] and other fields. At present, the preparation methods of UHMWPE fiber include solid-state hot drawing, solid-state extrusion, free growth and gel spinning [7]. Among them, since it has advantages of continuous processing and high processing efficiency, gel spinning is the most commonly used approach [8,9]. During gel spinning, the approach is characterized by UHMWPE being dissolved in solvents such as paraffin oil and decalin to prepare UHMWPE gel spinning solution. However, the solvent-extraction process features at great energy consumption, pollution and high processing cost [10].

Melt spinning, an economical fiber manufacturing technology with high efficiency, low emissions and low cost, has been widely used in the production of polymer fiber, such as polyamide fiber, polyester fiber and polyolefin fiber. [11]. Research on the preparation of UHMWPE fiber by melt spinning is helpful to realize the production of UHMWPE fiber with low cost and high efficiency. Nevertheless, UHMWPE melt typically presents a high melt viscosity and poor fluidity due to massive entanglement of molecular chain [12]. The as-spun filament extrude through the small-diameter spinneret flow passages would easily distort and fracture, bringing about uneven diameters and low drawing ratio in the subsequent hot drawing procedure [13,14]. Therefore, the key to improve the flowability of UHMWPE determines whether the UHMWPE fiber can be prepared by melt spinning method. One of the most universal ways to improve the fluidity of UHMWPE is to blend it with low-molecular weight polymer. To date, UHMWPE incorporated with low molecular weight polyethylene [15], linear low-density polyethylene [16], medium molecular weight polyethylene [17], polypropylene [18], etc. have been studied to expand the application of UHMWPE. Among them, high density polyethylene (HDPE) is widely used in melt spinning on account of its low viscosity and relatively good compatibility with UHMWPE. Despite the addition of HDPE improving the melting spin-ability of UHMWPE, the mechanical property of blend fiber still needs to be enhanced for actual application comparison with UHMWPE fiber by gel spinning.

Adding nanoparticles to UHMWPE is an effective method to improve the fluidity of materials and the mechanical property of final fiber products. Yeh et al. [19,20,21,22] have reported that single-layer nanotubes, nano-SiO_2_, nano-alumina and nano-cellulose can serve as efficient nucleation sites for the crystallization of UHMWPE in the gel spinning process. Zhang et al. [23] suggested that the UHMWPE solution added with nano-SiO_2_ can increase the crystallinity, reduce the grain size and significantly improved the mechanical property. Multiwalled carbon nanotubes were used to reinforce gel-spun UHMWPE fiber by Shilun et al. [24]. The tensile strength and ductility of the fiber were 18.8% and 15.4% higher than that of pure UHMWPE fiber, respectively. Tam et al. [25] added a small amount of TiO_2_ (0.1–1 wt%) to the UHMWPE/HDPE blend, so that the blend melt could be smoothly extruded through the capillary die without melt fracture. Zhang et al. [7] remarked that the addition of organic montmorillonite during the melt-spinning preparation of UHMWPE improved the fluidity yet without affecting the crystal structure of UHMWPE fiber in the drawing process. From the above research, UHMWPE nanocomposites fiber is mainly prepared by gel spinning, while little research has been done on melt spinning. In practice, different preparation methods lead to different microstructures, such as grain size, internal defects, crystallinity, etc. 

Given the lack of research on UHMWPE melt spinning, the role of nanoparticles in different stages for melt spinning is not clarified. In this work, we prepared UHMWPE/HDPE fiber incorporated with different content of nano-SiO_2_ by using self-designed single screw melt spinning equipment. Firstly, the effect of SiO_2_ on the rheological property of UHMWPE/HDPE blend is investigated. The impact of nanoparticles on the microstructure and mechanical property of the material is also investigated at the extrusion stage of the as-spun filament and the hot drawing stage of the fiber. The research contents of this work can guide the optimal material system of UHMWPE fiber reinforced by nanomaterial.

## 2. Experimental

### 2.1. Materials

**Figure 1:**
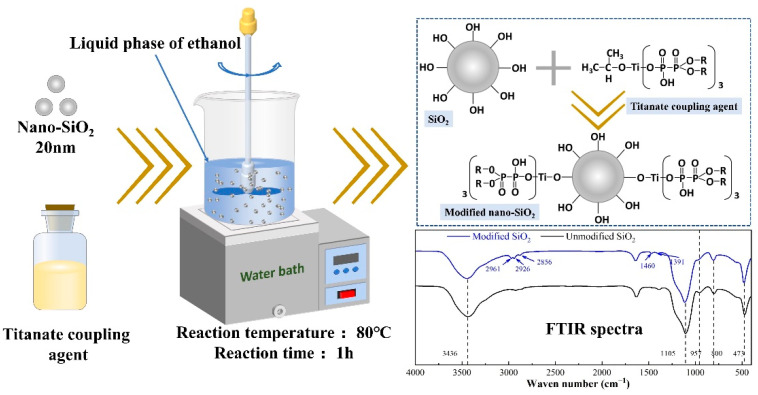
Schematic diagram for the surface modification process of nano-SiO_2_.

UHMWPE (GUR4012, molecular weight = 1.5 × 10^6^, powder state) was supplied by Nanjing Celanese Corporation, Nanjing, China. HDPE (grade-5070, powder state) with the melt flow index (MFI) of about 7.05 g/10 min (190 °C, 5 kg) and density of 0.958 g/cm^3^, was supplied by Liaoning Panjin Ethylene Co., Ltd., Panjin, China. Unmodified nano-SiO_2_ particles with average diameter of 20 nm were purchased from Shanghai Aladdin Biochemical Technology Co., Ltd., Shanghai, China. Generally speaking, nanoparticles were easy to aggregate into larger scale in the polymer matrix [26]. Surface treatment can effectively reduce the agglomeration of nano-fillers and facilitate its uniform dispersion in the polymer matrix [27]. Surface modification of SiO_2_ in this work was carried out by the titanate coupling agent (grade-38S, Feiyang Chemical factory, Jiangsu, China) in ethanol. The reaction temperature was 80 °C in the water bath and the reaction time was 1 h, as shown in Figure 1. Then, the surface modified nano-SiO_2_ was dried in a drying oven at 60 °C for 2 h to evaporate ethanol. In our previous research [28], we have found that the most appropriate mass ratio of UHMWPE to HDPE was 6:4, so the addition of nano-SiO_2_ with different contents is based on this. The mass percentages of nano-SiO_2_ were weighed according to the formulations listed in Table 1. Here, U, H and Si represent UHMWPE, HDPE and modified-nano-SiO_2_, respectively. The numerical suffix “−0.5, 1.0, 2.0” indicates that the modified nano-SiO_2_ content in nanocomposite is 0.5 wt%, 1.0 wt% and 2.0 wt%, respectively.

### 2.2. Preparation Procedures

**Figure 2:**
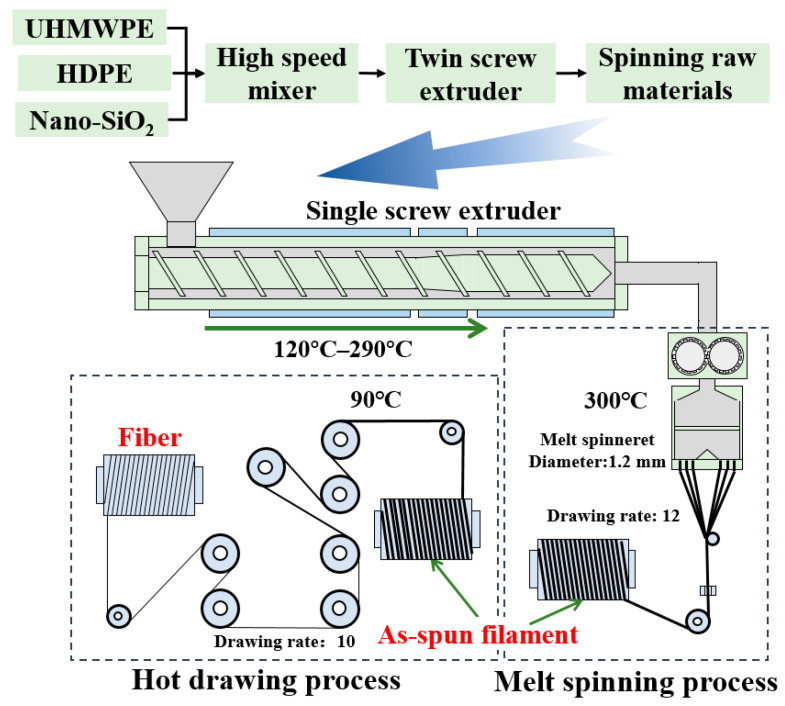
Preparation procedures of UHMWPE/HDPE/nano-SiO_2_ blend fiber.

UHMWPE, HDPE powder and modified-nano-SiO_2_ were blended in a high-speed mixer at room temperature with the rotational speed of 1440 rpm for 20 min. Then, these compositions were compounded and granulated using a corotating twin-screw extruder (aspect ratio L/D = 45 and screw diameter D = 20 mm). The screw speed was 190 rpm, and the temperature profile from the feed zone to the die zone was 160 to 290 °C. Melt spinning of the blended pellets was carried out by a self-made single-screw spinning apparatus. The initial draw ratio of as-spun filaments was about 12. The temperature of the screw extrusion section was 120 to 290 °C, and the temperature of the spinning pack with a spinneret orifice diameter of 1.2 mm was 300 °C. The hot drawing ratio was about 10 under 90 °C. The detailed preparation procedures are shown in Figure 2.

### 2.3. Characterizations and Measurements

Scanning electron microscope (SEM): The surface morphology and the tensile fracture cross section of as-spun filament and fiber were observed by the S4700 scanning electron microscopy (Hitachi Ltd., Tokyo, Japan) with the acceleration voltage of 20 kV.

Rheological measurements: Oscillatory shear measurements were performed using the MARS parallel-plate rheometer (HAAKE, Germany) under a nitrogen atmosphere. Measurements were conducted in parallel plates (25 mm diameter and 1 mm gap) with an angular frequency from 0.01 to 100 rad/s at 290 °C. The shear flow properties of composites were measured by the RH2000 piston-type capillary rheometer (Malvern, British), equipped with a 180° entry angle die having *L/D* = 16 and *D* = 1.0 mm. The shear rate range was 20–10,000 s^−1^, and the experiment temperature was 290 °C.

Tensile testing: Tensile testing of the fiber were measured using the YM-06B fiber electronic strength tester (Laizhou Yuanmao Instrument Co., Ltd., Laizhou, China) with a tensile speed of 20 mm/min. The test length of the fiber samples was 20 mm. At least eight samples were tested for each formulation to get the average.

Differential scanning calorimetry (DSC): The thermal properties of the as-spun filament and fiber were performed using the STARe System DSC2 differential scanning calorimeter (Mettler Toledo, Greifensee, Switzerland) in a nitrogen atmosphere. The heating rate of 10 °C/min and the temperature range of 70–180 °C were selected, and the crystallinity (*X_c_*) of all samples was calculated through Equation (1):

(1) $$X_{c}=\frac{\Delta H_{m}}{\Delta H_{m}^{0}}\times 100\%$$

The value of $\Delta H_{m}^{0}$ is the melting enthalpy for 100% crystalline PE, which is assigned as 292 J/g [29].

Wide-angle X-ray diffraction (WAXD): The WAXD measurements were carried out using the Bruker D8 Advance diffractometer (Bruker, Germany) with a scanning range of 2θ from 5° to 90° at the speed of 10 °/min. The crystal size *(L_hkl_*) perpendicular to the diffraction lattice planes was calculated from the half-height width of the crystalline reflections using the Scherrer equation:

(2) $$L_{hkl}=\frac{K\lambda }{\beta \cos\theta }$$

where *K* is the correction factor for lattice distortion (0.943); *λ* is the X-ray wavelength (1.542 Å); *β* is the half-height width of diffraction peak in radians; *θ* is the Bragg angle.

Sound velocity orientation testing: The orientation testing was carried out using SCY-II sound velocity orientation instrument (Shanghai Donghua Kelly company, Shanghai, China), and the sound velocity orientation factor of the sample was calculated by Equation (3):

(3) $$f_{s}=\left(1-\frac{C_{m}^{2}}{C^{2}}\right)\times 100$$

(4) $$C=\frac{L}{T_{2}-T_{1}}\left(\frac{\mathrm{km}}{s}\right)$$

where *C* is the sound velocity value of the sample; *L* is the distance between two measuring points; *T*_1_ and *T*_2_ are the time of sound passing through two measuring points; *C_m_* value of 1.65 km/h is the sound velocity of randomly oriented PE fiber. Generally, the higher of molecular chain orientation in polymer, the faster the propagation velocity of sound.

## 3. Results and Discussion

### 3.1. Rheological Property

**Figure 3:**
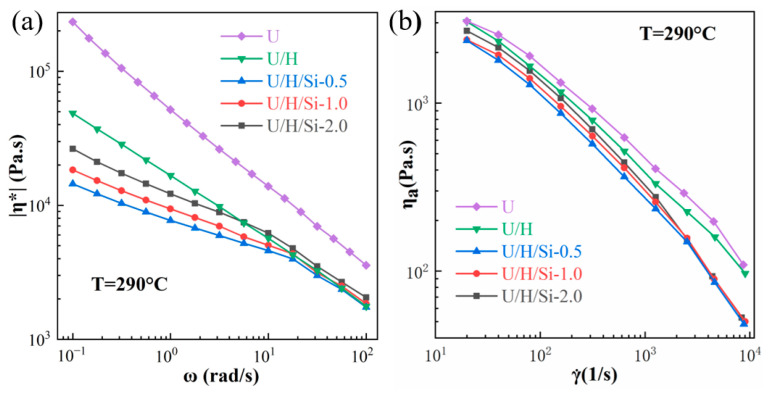
Rheological property of samples: (**a**) complex viscosity ($\left|\eta^{*}\right|$); (**b**) apparent viscosity ($\eta_{a}$ ).

Melt spinning is the process with high shear rates, and UHMWPE melt is prone to deformation and fracture due to high shear rates when passing small diameter spinneret channel [12]. Generally, the lower melt viscosity of polymer, the greater critical shear rate it can withstand, which makes melt rupture unlikely to occur during the extrusion process. Therefore, special attention should be paid to the rheological behavior of UHMWPE composites. Figure 3 shows the variation curves of complex viscosity with angular frequency and apparent viscosity with apparent shear rate for all samples at 290 °C. In comparison with U sample, the $\left|\eta^{*}\right|$ value of the U/H sample is obviously lower, and the same change appears in the apparent viscosity, indicating that the addition of HDPE facilitates the disentanglement of long molecular chain in UHMWPE. Meanwhile, these data suggest that the complex viscosity of all UHMWPE/HDPE/nano-SiO_2_ samples decreases compared to U/H sample, especially at low frequencies. Moreover, the complex viscosity of U/H/Si-0.5 is lower than that of U/H/Si-2.0 and U/H-Si-1.0. This phenomenon is also confirmed in Figure 3b, where the apparent viscosity of nanocomposites decreases with increasing shear rate at low shear rate stage, and there is a clear difference in viscosity. However, the viscosities of the nanocomposites all converge as the shear rates exceed 1000 s^−1^. Those behaviors suggested that the addition of nano-SiO_2_ particles improved the processability of UHMWPE/HDPE blend. This is because along with the shear action, the nano-SiO_2_ particles in molten nanocomposites may begin to slip, thus leading to favorable flow orientation, which subsequently reduced the viscosity of the PE matrix [30]. 

### 3.2. SEM Observation

**Figure 4:**
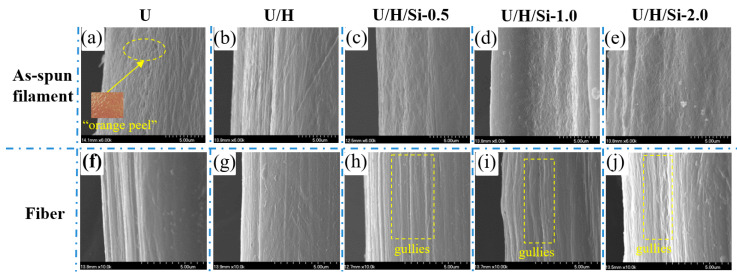
Surface micromorphology of samples: (**a**–**e**) as-spun filament samples of U, U/H, U/H/Si-0.5, U/H/Si-1.0 and UH/Si-2.0; (**f**–**j**) fiber samples of U, U/H, UH/Si-0.5, UH/Si-1.0 and UH/Si-2.0.

The surface micromorphology of as-spun filament and fiber samples are observed by SEM, as shown in Figure 4. It can be noticed from Figure 4a–e that the surface of each as-spun filament sample is irregular, and the texture similar to “orange peel” can be observed. Among them, the surface regularity of the U as-spun filament sample is the worst, while the U/H as-spun filament sample has the most regular surface morphology, where the texture oriented in the axial direction can be observed. The surface morphology of each fiber sample became more regular after the hot drawing process, as shown in Figure 4f–j. The orientation structure along the axial direction can be clearly observed, but there are also certain “gullies”. Especially, the surface orientation structure of the three nanocomposite fiber samples is more apparent, and the morphology appears to be denser, with U/H/Si-0.5 fiber being the most remarkable.

**Figure 5:**
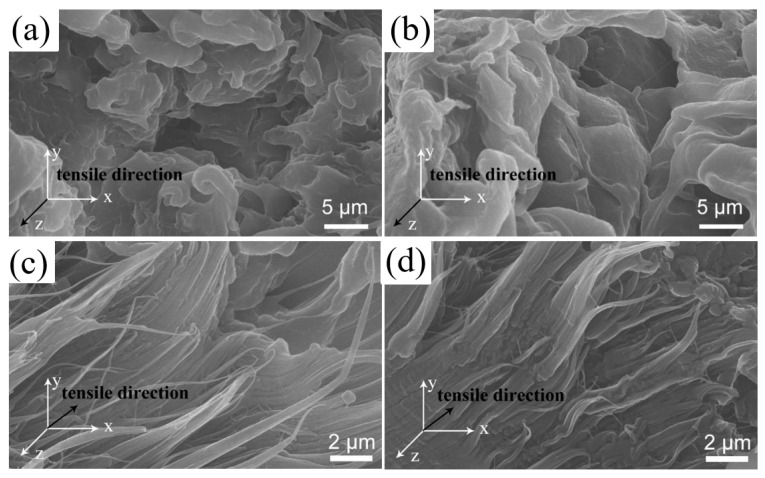
Micromorphology of tensile cross-section of samples: (**a**) U/H as-spun filament; (**b**) U/H/Si-0.5 as-spun filament; (**c**) U/H fiber; (**d**) U/H/Si-0.5 fiber.

The tensile cross-section micromorphology of U/H and U/H/Si-0.5 as-spun filament and fiber samples are shown in Figure 5. The cross-section microstructure of as-spun filament samples shows a layered morphology that peels off from each other along the axial direction. It can be noted from Figure 5c,d, after the hot drawing process, the micro-fibril structure orderly arraying along the axial direction can be observed in the fiber samples. This indicates that microfibrillar structures are indeed generated within the UHMWPE fiber that contribute to the mechanical property.

### 3.3. Crystallization and Molecular Chain Orientation

**Figure 6:**
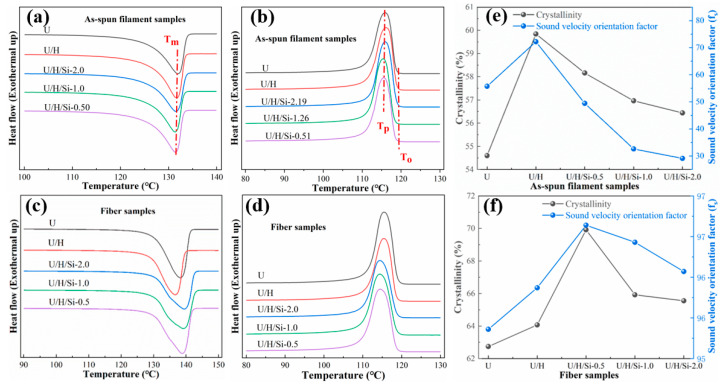
(**a**–**d**) DSC curves of all as-spun filament and fiber samples; (**e**) Crystallinity and sound velocity orientation factor of as-spun filament samples; (**f**) Crystallinity and sound velocity orientation factor of fiber samples.

The melting and crystallization behaviors of all as-spun filament and fiber samples are characterized by DSC. The sound velocity orientation factor (f_s_) is used to indicate the degree of molecular chain orientation in as-spun filament and fiber, which is positively correlated with the degree of molecular chain orientation [31]. Figure 6a–f show the thermal properties, crystallinity and sound velocity orientation factors (f_s_) of all samples.

According to the listed DSC curves, there is only one peak in the heating melting and cooling crystallization curves of all as-spun filament and fiber samples, which suggest that partial co-crystallization occurred in UHMWPE/HDPE [32], and the co-crystallization process is hardly affected by SiO_2_. Table 2 lists the peak crystallization temperature (T_p_) and onset crystallization temperature (T_o_) of as-spun filament samples from DSC curves. Compared with U/H sample, the T_p_ and the T_o_ of the nanocomposites as-spun filament samples are slightly moved to lower temperature. The increase in $T_{o}-T_{p}$ of the nanocomposite samples compared to U/H suggests a minor reduction in the crystallization rate [33]. These phenomena illustrate that the addition of nano-SiO_2_ does not induce heterogeneous nucleation in UHMWPE/HDPE as-spun filament by melt spinning.

As can be seen from Figure 6e, the molecular chain orientation of all nanocomposite as-spun filament samples is lower than that of the U/H as-spun filament samples, and reduces with the addition of nano-SiO_2_ content. Additionally, the crystallinity of as-spun filament decreased from 58.16% with 0.5 wt% nano-SiO_2_ to 56.44% with 2.0 wt% nano-SiO_2_. Causing this effect may be the presence of SiO_2_ nanoparticles in the form of entangled nodes during the melt-spinning process, which restricts the orientation of molecular chain. In general, the lower the molecular chain orientation in the polymer, the lower the crystallinity [34,35]. With the addition of SiO_2_ nanoparticles, the number of entanglement nodes increases, which results in lower molecular chain orientation and crystallinity of as-spun filament. From the Figure 6c,d, it can be seen that melting and crystallization curve peaks of the fiber samples are wider than that of as-spun filament samples, indicating that the crystal size distribution in each fiber samples may be lightly broader. 

Under the synergistic effect of high-rate hot drawing and SiO_2_ nanoparticles, the crystallinity and molecular chain orientation of nanocomposite fiber samples are higher than those of U/H fiber samples, as shown in Figure 6f. This implies that some molecular chain segments are stretched and arranged in a regular order to form the crystal lattice in the amorphous and defective crystalline regions. The highest molecular chain orientation and crystallinity (67.92%) in the fiber is achieved when the nano-SiO_2_ is incorporated at 0.5 wt%. Nevertheless, it is notable that the crystallinity of nanocomposite fiber reduces with increasing nano-SiO_2_ content, yet all are higher than that of UHMWPE/HDPE fiber. The trend of molecular chain orientation is the same as the trend of crystallinity.

**Figure 7:**
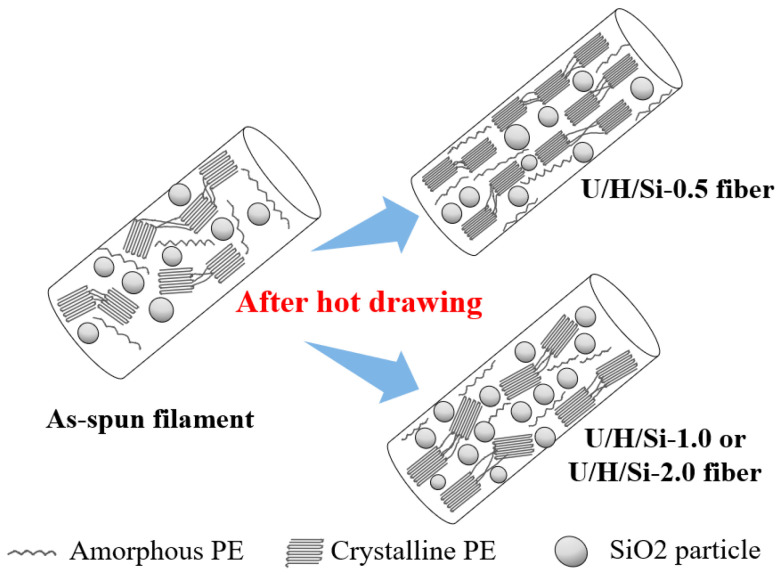
Possible mechanism of modified nano-SiO_2_ in hot drawing stage.

The potential mechanism interpretation for the effect of SiO_2_ nanoparticles on fiber crystallinity and molecular chain orientation is shown in Figure 7. In the hot drawing process, the cross-sectional dimension and volume of the fiber are reduced. The nanoparticles with low content are evenly dispersed in the amorphous region, functioning as the “lubricant”, so as to achieve the orderly orientation of molecular chain segments in the amorphous region to produce more crystalline regions [24,36]. Moreover, it promotes a more orderly and compact arrangement of existing crystals. On the contrary, when the content is high, evenly dispersed nanoparticles in the amorphous region gradually aggregate and agglomerate with decreasing cross-section. Here, nanoparticles played stronger role as physical barrier than as “lubricant”. It is distributed around the crystal as physical barrier, restricting the further improvement and rearrangement of the crystal. Simultaneously, it restricts the ordered orientation of molecular fragments located in the amorphous region.

### 3.4. Grain Size

**Figure 8:**
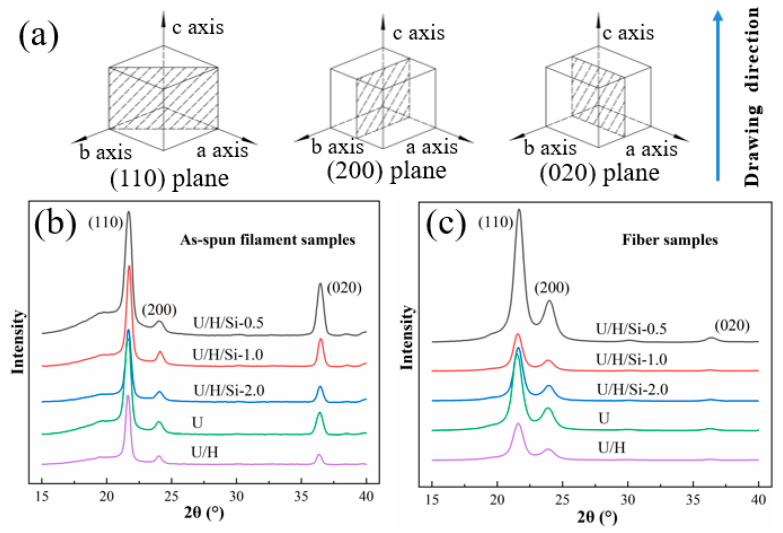
(**a**) © schematic diagram of three diffraction peaks; (**b**) WAXD patterns of as-spun filament samples; (**c**) WAXD patterns of fiber samples.

The internal crystallization unit of PE fiber prepared by melting method existed in the form of orthogonal crystals, and the schematic diagram of three diffraction planes: the (110), (200) and (020) is shown in Figure 8a. The WAXD patterns of as-spun filament and fiber samples are shown in Figure 8b,c.

As can be seen, all as-spun filament and fiber samples have diffraction peaks near 2θ = 21.6°, 24°, and 36.3°, corresponding to the (110), (200), (020). and (002) diffraction planes, respectively. The phenomenon indicates that the addition of modified-nano-SiO_2_ does not cause lattice distortion in the crystalline region.

The average grain size of the U/H-40 as-spun filament sample is greater than that of the U filament sample, as shown in Table 3. Because the short HDPE molecular chain increase the spacing of UHMWPE molecular chain, which reduces the entanglement points between its molecular chain and contribute to the growth of grain size. The average grain size of nanocomposites as-spun filament samples is smaller than that of U/H as-spun filament samples. This is due to the fact that the interfacial adhesion is still present in the nanocomposites, while the nano-SiO_2_ acts as entanglement points, limiting the movement of the macromolecular chain and resisting the growth of the grain during the extrusion [32,37]. The average grain size of all nanocomposite fiber is larger than that of U/H fiber. For the same reason as the effect of nanoparticles on the crystallinity and orientation of fiber. The nanoparticles function as “lubricators” during the hot drawing process, rearranging the crystals and refining the defective ones, thus promoting the crystals growth. However, with the addition of SiO_2_, some nanoparticles were aggregated around the crystals, which hinder the crystal refinement process, without further development of grain size.

### 3.5. Mechanical Property

**Figure 9:**
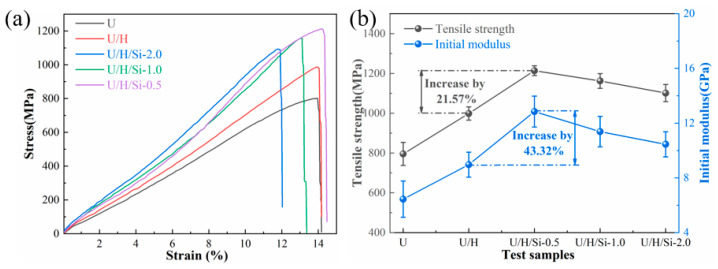
(**a**) Stress-strain curves of all fiber samples; (**b**) tensile strength and initial modulus for all fiber samples.

The typical stress-strain curves, tensile strength and initial modulus for U, U/H, U/H/Si-0.5, U/H/Si-1.0 and U/H/Si-2.0 fiber samples are showed in Figure 9**.** Basically, the high strength property of UHMWPE fiber are the consequences of the deformation mechanism, which entail a better orientation of the molecular chain during the hot drawing process [38]. Actually, the strength of materials is limited by the presence of flaws and cracks [39]. For composites fiber containing nano-SiO_2_, the dispersion of the nanoparticles and the interfacial adhesion to the polymer matrix determine their reinforcement in the PE matrix [30,40]. The stress-strain curves in Figure 9a do not have good similarity in shape and obvious regularity. It is judged that the structure of the fibers prepared by melt spinning method still has some defects.

Figure 9b shows the tensile strength and initial modulus of all fiber samples. The tensile strength and initial modulus of U melt-spun fiber samples were 796.2 MPa and 6.45 GPa, respectively. While the nanocomposite fiber incorporating 0.5 wt% nano-SiO_2_ displays the maximum tensile strength and initial modulus with 1095.46 MPa and 10.42 GPa, respectively. This means that the addition of a low amount of nano-SiO_2_ can strengthen the fiber, while the continuously increasing filler content is detrimental to the mechanical property of UHMWPE/HDPE fiber. In fact, the degree of molecular chain orientation and crystallinity are the main factors for affecting the mechanical property of polymer fiber. According to the analysis of crystallization behavior and molecular chain orientation in the previous chapter, we know that the addition of 0.5 wt% nano-SiO_2_ not only contributes to the improvement of crystallinity and molecular chain orientation, but also facilitates the perfection and fineness of the grains., which is reflected in the remarkable improvement of mechanical property.

## 4. Conclusions

The purpose of this work is to investigate the impact of nano-SiO_2_ incorporation on the crystal structure, molecular chain orientation of UHMWPE/HDPE during melt spinning (spinning stage and hot drawing stage), and the mechanical characteristics of the produced fiber. Furthermore, the effect of nano-SiO_2_ incorporation on the viscosity of blend is also investigated. The main conclusions are as follows.

(1) The incorporation of nano-SiO_2_ improves the flowability of the UHMWPE/PE blend. However, under the effect of high shear rate, the content of nano-SiO_2_ had no obvious effect on the viscosity for matrix material, the apparent viscosity and the complex viscosity tends to be the identical.
(2) The addition of nano-SiO_2_ restrains the crystallization and molecular chain orientation in the as-spun filament, and both reduces with the addition of nano-SiO_2_ content. After hot drawing, the crystallinity and molecular chain orientation of nanocomposite fiber are higher than those of UHMWPE/PE fiber and are most obvious when the nano-SiO_2_ content is 0.5 wt%.
(3) In the extrusion stage of as-spun filament, the addition of nano-SiO_2_ increase the entanglement points of the molecular chain, which caused the as-spun filament grain size of nanocomposite to be smaller than that of the UHMWPE/HDPE as-spun filament. However, after hot drawing, the nano-SiO_2_ have a promotion effect on grain refinement and the grain size becomes larger, continuing the addition of nanoparticles does not have a significant promotion effect.
(4) Since both the crystallinity and molecular chain orientation are improved and the grain size is refined, when the content is 0.51 wt%, UHMWPE/HDPE/modified nano-SiO_2_ shows the best mechanical property with tensile strength and initial modulus of 1211 MPa and 12.81 GPa, respectively.

## Figures and Tables

**Table 1:** The formulation of UHMWPE, HDPE and nano-SiO_2_ in different samples.

Sample	Mass Ratio of UHMWPE/HDPE	Content of Nano-SiO_2_ (wt %)
**U**	1	0
**U/H**	6/4	0
**U** **/H/Si-0.5**	6/4	0.5
**U** **/H/Si-1.0**	6/4	1.0
**U** **/H/Si-2**	6/4	2.0

**Table 2:** Thermal property characteristics of U, U/H, U/H/Si-0.5, U/H/Si-1.0, and U/H/Si-2.0 as-spun filament samples.

As-Spun Filament Sample	$T_{p}$ (°C)	$T_{o}$ (°C)	$T_{o}-T_{p}$ (°C)	$T_{m}$ (°C)	$\Delta T=T_{m}-T_{p}$ (°C)
U	115.9	118.62	2.72	131.97	16.07
U/H	116.12	118.79	2.67	131.67	15.55
U/H/Si-0.5	115.55	118.38	2.83	131.43	15.88
U/H/Si-1.0	115.41	118.43	2.83	131.36	15.95
U/H/Si-2.0	115.94	118.79	2.85	131.57	15.63

**Table 3:** Grain sizes of all as-spun filament and fiber samples.

As-Spun Filament Samples	Grain Size(nm)				Fiber Samples	Grain Size(nm)			
	110 Plane	200 Plane	020 Plane	Average		110 Plane	200 Plane	020 Plane	Average
**U**	15.4	13.0	15.1	14.5	**U**	9.7	8.0	7.9	8.5
**U/H**	20.2	18.4	19.4	19.3	**U/H**	9.5	7.6	10.0	9.0
**U/H/Si-0.5**	16.9	13.4	16.3	15.5	**U/H/Si-0.5**	10.2	8.2	9.1	9.2
**U/H/Si-1.0**	17.2	14.1	17.0	16.1	**U/H/Si-1.0**	10.6	8.4	9.2	9.4
**U/H/Si-2.0**	17.6	14.3	17.4	16.4	**U/H/Si-2.0**	10.7	8.5	8.7	9.3

## Data Availability

Not applicable.
